# Organizational features of mental health facilities and adequacy of treatment. Multilevel analysis from the italian region lombardy

**DOI:** 10.1192/j.eurpsy.2023.410

**Published:** 2023-07-19

**Authors:** C. Conflitti, M. Monzio Compagnoni, A. Lora, G. Corrao

**Affiliations:** ^1^Statistics and Quantitative Methods, University of Milan - Bicocca, Milan; ^2^Department of Mental Health, ASST Lecco, Lecco; ^3^National Centre of Healthcare Research & Pharmacoepidemiology, Milan, Italy

## Abstract

**Introduction:**

Mental healthcare represents a significant issue for the Healthcare System [1]: one of the major causes is the high heterogeneity in the provision of care, due to the differences among the Departments of Mental Health (DMHs).

**Objectives:**

To identify the predictors of an adequate treatment for patients with severe mental disorders (SMDs), both at an individual and structural level, the latter considering the major features of the Psychiatric Operative Units (POUs), in the Italian region Lombardy.

**Methods:**

Healthcare Utilization Databases, collecting data on the services provided to beneficiaries of the National Health Service (NHS), have been used to retrieve data.

Patients that during 2015 were resident in Lombardy; suffered from depressive, bipolar or schizophrenic disorder; were in contact with the DMHs, have been identified.

Adequateness of treatment has been evaluated according to the Minimally Adequate Treatment (MAT) [2,3]: a combination of psychiatric visits and specific pharmacological treatment, or psychotherapeutic sessions. Having received a MAT has been assessed during a one-year period.

Predictive factors have been classified into two hierarchical levels: individual (first) level and structural (second) level. At the first level, clinical and socio-demographics characteristics have been evaluated for each patient. At the second level, the organizational structure of each POU has been examined: the number of patients taken in care, number of community-care facilities, and hours worked by each class of healthcare providers (psychiatrists, nurses, psychologists, psychosocial staff ).

A log-binomial model has been used to evaluate the association between the first-level factors and having received a MAT; a multilevel log-binomial model for the second-level factors considered the hierarchical structure of data.

**Results:**

72115 patients have been identified: 33974 (47.1%), 28407 (39.4%) and 9734 (13.5%) suffering from depressive, schizophrenic or bipolar disorder respectively; 45.4% of them (32773 patients) received a MAT.

Compared with patients affected by depression, those with bipolar or schizophrenic disorder show a higher probability of receiving a MAT (+23%, +11% respectively).

Patients living alone have a lower probability of receiving a MAT, unlike patients with a higher level of education or employment, underlining the social burden related to SMDs.

Organizational features have proven significant: centres with a higher activity volume and with more community-care facilities seem more likely to guarantee MAT. Moreover, the higher the hours worked by psychiatrists, nurses and psychologists, the higher the probability of providing MAT.

**Conclusions:**

Patients with SMDs are still untreated in an appropriate way. Results highlight the importance of the community-care facilities, as well as of the composition of the multidisciplinary teams working there.

**Disclosure of Interest:**

None Declared

